# A phase 2, double-blind, placebo-controlled study of NSI-189 phosphate, a neurogenic compound, among outpatients with major depressive disorder

**DOI:** 10.1038/s41380-018-0334-8

**Published:** 2019-01-09

**Authors:** G. I. Papakostas, K. Johe, H. Hand, A. Drouillard, P. Russo, G. Kay, R. Kashambwa, B. Hoeppner, M. Flynn, A. Yeung, M. A. Martinson, M. Fava

**Affiliations:** 1 Massachusetts General Hospital Clinical Trials Network and Institute (MGH CTNI), Boston, MA USA; 2 Neuralstem, Inc, Germantown, MD USA; 3 Cognitive Research Corp., Saint Petersburg, Florida USA

**Keywords:** Depression, Drug discovery

## Abstract

NSI-189 is a novel neurogenic compound independent of monoamine reuptake pathways. This trial evaluated oral NSI-189 as monotherapy in major depressive disorder. To improve signal detection, the sequential-parallel comparison design (SPCD) was chosen. Two hundred and twenty subjects were randomized to NSI-189 40 mg daily, 80 mg daily, or placebo for 12 weeks. The primary outcome measure was the Montogmery Asberg Depression Rating Scale (MADRS). Secondary subject-rated measures included the Symptoms of Depression Questionnaire (SDQ), the Cognitive and Physical Functioning Scale (CPFQ), the patient-rated version of the Quick Inventory of Depressive Symptomatology Scale (QIDS-SR), and subtests from the CogScreen and Cogstate cognitive tests. MADRS score reduction versus placebo did not reach significance for either dose (40 mg pooled mean difference −1.8, *p* = 0.22, 80 mg pooled mean difference −1.4, *p* = 0.34, respectively). However, the 40 mg dose showed greater overall reduction in SDQ (pooled mean difference −8.2; Cohen’s *d* for Stages 1 and 2 = −0.11 and −0.64, *p* = 0.04), and CPFQ scores (pooled mean difference −1.9; Cohen’s *d* for Stages 1 and 2 = −0.28 and −0.47, *p* = 0.03) versus placebo, as well as QIDS-SR scores in Stage 2 of SPCD (−2.5; Cohen’s *d* Stages 1 and 2 = −0.03 and −0.68, *p* = 0.04). The 40 mg dose also showed advantages on some objective cognitive measures of the CogScreen (absolute Cohen’s *d* ranged between 0.12 and 1.12 in favor of NSI-189, *p* values between 0.002 and 0.048 for those with overall significance), but not the Cogstate test. Both doses were well tolerated. These findings replicate those of phase 1b study, and warrant further exploration of the antidepressant and pro-cognitive effects of NSI-189.

## Introduction

Major depressive disorder (MDD) is a highly prevalent medical illness [1], often associated with significant morbidity, mortality, and functional impairment [2, 3]. For many patients suffering from MDD, treatments delivered do not always have the desired effect [4–6]. Therefore, it remains crucial for the field to aid in the development of new and, most importantly, novel antidepressants [7–11].

To date, drug development for depression has been largely based on screening for agents with an affinity for monoamine transporters, then testing these agents preclinically using a rather limited battery of animal models such as the forced-swim test and the tail suspension test [12] before antidepressant efficacy is determined in phase 2 and 3 trials. More recently, in order to accelerate and diversify the discovery of new treatment strategies for depression, a different approach has been to utilize pre-clinical platforms based on existing neurobiological evidence for potential down-stream effects of antidepressants.

One such assay involves the measurement of hippocampal (HI) neurogenesis, since adult HI neurogenesis is believed to play a salient and direct role in the down-stream therapeutic effects of antidepressants [13], while chronic MDD had been linked to a loss of HI volume [14, 15]. For instance, a recent large meta-analysis of 1728 MDD subjects versus 7199 controls found that HI was the most pronounced brain area with volume shrinkage and that such HI atrophy was greatest in patients with recurrent episodes of MDD and in those with early onset of first episode [16]. In addition, HI volume reduction in MDD may be linked to reduced dentate neurogenesis specifically as well as more broadly to reduced synaptogenesis [17]. Thus, MDD symptomology may precipitate from reduced HI neurogenesis/synaptogenesis that results in distorted HI network structurally.

Cognitive impairment is an integral component of MDD symptomology. However, almost all existing antidepressants are not particularly effective against cognitive dysfunction [18–21]. In addition to mood dysregulation, the role of HI neurogenesis in cognitive impairment has been widely studied in general [22, 23]. Current evidence suggests that inhibition of HI neurogenesis may also be responsible specifically for the cognitive impairment in depressed patients [24, 25]. Hence, treatments that stimulate HI may be better suited for targeting cognitive symptoms in MDD.

NSI-189 is a novel, neurogenic compound independent of serotonin or norepinephrine reuptake inhibition pathways. The compound was discovered by systematic screening of a chemical library against in vitro model of HI neurogenesis using a stable cell line of human fetal HI neural stem cells (US Pat. No. 8,293,488; 7,650,533). Subsequently, its neurogenic activity was validated in vivo by increasing dentate gyrus neurogenesis in healthy normal young adult mice. Unique from other such neurogenic “hits” from the same screening campaign, NSI-189 also increased HI volume in healthy normal young adult mice [26]. Most importantly, the results of a small (*n* = 24) phase 1b, double-blind, randomized, placebo-controlled, multiple-dose study in MDD inpatients demonstrated a promising greater reduction in depressive as well as cognitive symptoms for NSI-189 than placebo [27]. The goal of the present study was to evaluate the efficacy, safety, and tolerability of two different doses of oral NSI-189 as monotherapy in outpatients with MDD. For this purpose and to optimize signal detection [10, 28], the sequential-parallel comparison design (SPCD) was chosen.

## Methods

This study was a 12-week, randomized, double-blind, SPCD [28–30] trial of NSI-189 monotherapy for MDD (ClinicalTrials.gov registration number NCT02695472). The study screened 353 recurrent MDD patients and randomized 220 subjects from 12 US, non-academic sites over an approximate 9-month period. The detailed study schedule of activities is shown in Supplemental Table 1. Institutional review board-approved written informed consent was obtained from all study patients before any study procedures were conducted. Eligibility was assessed during a site screen visit, followed by a remote assessment involving the SAFER interview conducted by Massachusetts General Hospital Clinical Trials Network and Institute (MGH CTNI) clinicians [31, 32] and, finally, by a site baseline visit. Safety was monitored by an independent MGH CTNI clinician who served as medical monitor for the study (GIP); a data safety and monitoring board was not involved.

Patient inclusion and exclusion criteria were as follows:

*Inclusion criteria*: Patients were eligible for study participation if they were between the ages of 18–60 years, with current MDD of at least 8 weeks duration according to the fifth version of the Diagnostic and Statistical Manual for Mental Disorders (DSM-5), as diagnosed by the Structured Clinical Interview for the DSM-5 clinical trial version (SCID-5-CT) (http://scid5.org/info/) during the screen and remote assessment visits, and if they were scored at least 20 at screen, remote assessment, and baseline visits on the Montgomery-Asberg Depression Rating Scale (MADRS [33])

*Exclusion criteria*: The following patients were excluded from being randomized in the study: (1) pregnant or lactating women or women with a positive serum or urine pregnancy test administered at screening and baseline, (2) women of childbearing potential not on a medically acceptable form of birth control or who did not agree to continue such birth control for the duration of the study, (3) clinically significant history or evidence of cardiovascular, respiratory, hepatic, renal, gastrointestinal, endocrine, neurological, immunological, or other major disease as determined by the site investigator such that participation in the study would place the subject at increased risk for a serious adverse event, (4) lifetime history of mania, hypomania, or psychosis, (5) a primary psychiatric diagnosis currently other than MDD, (5) non-response to at least three antidepressant trials of adequate dose and duration during the current major depressive episode as defined by the MGH Antidepressant Treatment History Questionnaire (MGH-ATRQ; Fava, 2003 [34]) and administered both by site investigators and MGH CTNI raters remotely (meeting this criterion during either assessment was sufficient for exclusion), (6) subjects with significant suicidal ideation, (7) subjects with an alcohol or drug use disorder active within the past 12 months, or a positive urine drug screen for drugs of abuse at either screening or baseline, and (8) patients on an excluded medication (antidepressants, antipsychotics, buspirone, and lithium were excluded, while anticonvulsants, dopamine agonists, psychostimulants, modafinil, T3, benzodiazepines, zolpidem, zaleplon, eszopiclone, melatonin, and low-dose trazodone were allowed if stable in dose for at least 4 weeks prior to the screen visit).

### Study procedures

Enrolled patients were randomized in a 1:1:3 fashion to receive fixed-dose treatment with NSI-189 40 mg daily during Stages 1 and 2, NSI-189 80 mg daily during Stages 1 and 2, or to receive placebo during Stage 1. The higher probability of randomization to placebo in Stage 1 is to generate a sufficient sample size for Stage 2 (since Stage 1 placebo non-responders comprise the entire Stage 2 efficacy sample). Hence, placebo-treated patients who completed Stage 1 and met the specific criteria for non-response (see below) were then re-randomized in a 1:1:1 fashion to receive either placebo, NSI-189 40 mg daily, or NSI-189 80 mg daily during Stage 2. Specifically, the criteria for re-randomization were as follows: (1) <50% reduction in MADRS scores from baseline during Stage 1 and (2) MADRS score >15 during the baseline visit of Stage 2. The sixth post-randomization visit served as the final visit for Stage 1, the re-randomization visit for placebo-treated subjects who completed Stage 1 and who met criteria the criteria listed above, and the baseline visit for Stage 2. The following scales were administered during the randomization and several (either 6 or 8) post-randomization visits: MADRS, the clinician-rated 17-item Hamilton depression rating scale (HAMD-17 [35]), clinical global impressions-severity and improvement (CGI-S/I [36]), the self-rated MGH Cognitive and Physical Functioning Questionnaire (MGH CPFQ [37]), and the self-rated Symptoms of Depression Questionnaire (SDQ [38]), which also includes the seven items of the CPFQ. The self-rated version of the quick inventory for depressive symptomatology (QIDS-SR [39]) was administered during the randomization, re-randomization, and study final visits (end of Stage 2).

### Objective cognitive measures

In addition to the CPFQ, two objective cognition instruments were used in the study: Cogstate and CogScreen. Both were administered using a computer interface. The Cogstate battery consisted of the following tests: (1) Detection, (2) Identification, (3) One Card Learning, and (4) One Back. The CogScreen battery consisted of the following subtests: (1) Previous Number Alone, (2) Shifting Attention Test Arrow Direction, (3) Shifting Attention Test Arrow Color, (4) Shifting Attention Test Instruction, (5) Shifting Attention Test Discovery, (6) Symbol Digit Coding, and (7) Symbol Digit Coding Delayed Recall.

### General statistical considerations

Efficacy analysis was performed on the full analysis set (FAS) who received at least one dose of study drug at the visit subsequent to randomization (or re-randomization in Stage 2), and had at least one post-randomization (or re-randomization in Stage 2) MADRS assessment. Safety evaluation was performed on all subjects randomized in the study who received at least one dose of study drug (safety dataset), separately for Stages 1 and 2.

The primary efficacy analysis used Stage 1 and 2 FAS using a mixed model repeated measures (MMRM). The effect within each treatment was measured as the change in the MADRS total score from baseline to the end of treatment, and was calculated by stage. An unstructured variance/covariance structure was used for the statistical modeling. The weighted restricted maximum likelihood (REML) estimate for differences between active and placebo groups was calculated for each active treatment group for each stage. In order to account for multiplicity due to the two pairwise comparisons of NSI-189 (80 and 40 mg) versus placebo, the sequentially rejective Hommel procedure [40] was applied to all overall *p* values with the exception of CogScreen and Cogstate, which were exploratory analyses. An analysis of covariance (ANCOVA) model was used to assess the sensitivity of the primary analysis of MADRS to statistical assumptions behind the MMRM. Responder and remitter analyses were performed stage wise using the logistic regression model to assess the robustness of the results. All analyses described for the primary efficacy endpoint were also applied for the HAMD17, SDQ, and MGH CPFQ. The CGI-S and CGI-I were analyzed using the FAS for each stage using a proportional odds logistic regression. As an exploratory analysis, the frequencies of CGI-I and CGI-S outcome were summarized by visit for all subjects who received the same treatment in both stages.

*Safety analyses*: All safety parameters were summarized separately for Stages 1 and 2 as well as overall, using the safety set. Differences in the incidence of treatment emergent adverse events (TEAEs) between treatment groups were presented using descriptive statistics. A summary of TEAEs was also presented by Medical Dictionary for Regulatory Activities system organ class and preferred term. Vital sign measurements, electrocardiogram results, laboratory assessments, and physical examination findings were presented using descriptive statistics.

*Analysis of efficacy (SPCD)*: The SPCD test statistic is based on a weighted combination of the estimated treatment effects [41]. The treatment effects in Stage 1 and the variances of the estimated treatment effect were obtained from a linear mixed model. The treatment effects in Stage 2 and their variances were estimated in a similar way. Only data from Stage 1 placebo non-responders who were re-randomized were used to estimate Stage 2 treatment effects. The treatment estimates were weighted means of the estimated effects from the two stages: $\widehat {\theta _j} = w\widehat \theta _{1,j} + \left( {1 - w} \right)\widehat \theta _{2,j}$, and the variance of the treatment estimate under the null hypothesis is ${\mathrm{Var}}( {\widehat {\theta _j}} ) = w^2{\mathrm{Var}}( {\widehat \theta _{1,j}} ) + ( {1 - w} )^2{\mathrm{Var}}( {\widehat \theta _{2,j}} )$. Here *j* = 1,2 denotes the 40 day and 80 mg/day groups. Note that ${\mathrm{Var}}( {\widehat \theta _{1,j}} ),\;j = 1,2$ is simply the square of the standard error estimates of the treatment effects given in the statistical output of the MMRM.

Estimated treatment effects and their variances were then combined into the SPCD test statistic:

$$T_j = \frac{{w\widehat \theta _{1,j} + \left( {1 - w} \right)\widehat \theta _{2,j}}}{{\sqrt {w^2{\mathrm{Var}}\left( {\widehat \theta _{1,j}} \right) + \left( {1 - w} \right)^2{\mathrm{Var}}\left( {\widehat \theta _{2,j}} \right)} }},\; j = 1,2.$$

*T*_*j*_ is asymptotically standard normally distributed and therefore the *p* values were computed as *p*_*j*_ = 2(1 − *Φ*(|*T*_*j*_|)) for *j*=1,2, there *Φ*(*x*) is the standard normal cumulative distribution function [41]. For this study, *w* = 0.5.

The 95% confidence interval for the overall treatment effect was calculated as:

$$	\left(\vphantom{\sum^{x}}\right.{w\hat \theta _{1,j} + \left( {1 - w} \right)\hat \theta _{2,j} - {\mathrm{Z}}_{0.025}}\\ 	\hskip 10pt \cdot \sqrt {w^2{\mathrm{Var}}\left( {\hat \theta _{1,j}} \right) + \left( {1 - w} \right)^2{\mathrm{Var}}\left( {\hat \theta _2} \right)} ,\\ 	w\hat \theta _{1,j} + \left( {1 - w} \right)\hat \theta _{2,j} + {\mathrm{Z}}_{0.025}\\ 	\hskip 10pt { \cdot \sqrt {w^2{\mathrm{Var}}\left( {\hat \theta _{1,j}} \right) + \left( {1 - w} \right)^2{\mathrm{Var}}\left( {\hat \theta _{2,j}} \right)} } \left.\vphantom{\sum^{x}}\right).$$

For each comparison of dose group versus placebo, the sum of the equally weighted stage-wise weighted REML difference estimates and the corresponding test statistics were used to summarize the estimates and perform inference integrating the data from the two stages.

## Results

Two hundred and twenty subjects were randomized to receive treatment with NSI-189 40 mg daily (*n* = 44) during Stages 1 and 2, NSI-189 80 mg daily (*n* = 44) during Stages 1 and 2, or to receive placebo during Stage 1 (*n* = 132). In the latter group, 107 (approximately 81%) subjects completed Stage 1, of which 41 (approximately 38.3%) were classified as placebo treatment responders and 66 as placebo non-responders. Placebo non-responders were then re-randomized in a 1:1:1 fashion to receive either placebo (*n* = 22), NSI-189 40 mg daily (*n* = 22), or NSI-189 80 mg daily (*n* = 22) in Stage 2. A total of 170 subjects (77% of randomized) completed the 12-week treatment period. Further details on subject disposition are reported in Supplemental Figure 1, and baseline demographic/clinical variables in Table 1.

**Table 1:** Baseline demographic and clinical variables: FAS

	NSI-189 40 mg/day	NSI-189 80 mg/day	Placebo
Age in years, mean (s.d.)			
Stage 1	38.0 (10.2)	42.3 (12.0)	42.3 (10.9)
Stage 2	45.3 (9.6)	42.9 (11.2)	44.3 (11.2)
Gender—women, *N* (%)			
Stage 1	27 (62.8)	24 (55.8)	78 (62.9)
Stage 2	14 (66.7)	13 (59.1)	14 (63.6)
Race, *N* (%)			
Stage 1			
Caucasian	31 (72.1)	32 (74.4)	78 (62.9)
African American	11 (25.6)	9 (20.9)	38 (30.6)
Asian	1 (2.3)	1 (2.3)	2 (1.6)
Native American/Alaskan	0	0	0
Native Hawaiian/Pacific	0	0	0
Other	0	1 (2.3)	6 (4.8)
Stage 2			
Caucasian	12 (57.1)	17 (77.3)	12 (54.5)
African American	7 (33.3)	4 (18.2)	5 (22.7)
Asian	0	0	2 (9.1)
Native American/Alaskan	0	0	0
Native Hawaiian/Pacific	0	0	0
Other	2 (9.5)	1 (4.5)	3 (13.6)
Ethnicity, *N* (%)			
Stage 1			
Hispanic/Latino	15 (34.9)	5 (11.6)	20 (16.1)
Non-Hispanic/Latino	28 (65.1)	38 (88.4)	104 (83.9)
Stage 2			
Hispanic/Latino	3 (14.3)	5 (22.7)	3 (13.6)
Non-Hispanic/Latino	18 (85.7)	17 (77.3)	19 (86.4)
BMI (kg/m^2^), mean (s.d.)			
Stage 1	29.7 (4.9)	30.5 (4.2)	29.4 (4.8)
Stage 2	30.3 (3.5)	29.9 (4.5)	29.2 (4.7)
MADRS score, mean (s.d.)			
Stage 1	32.2 (5.5)	31.3 (5.6)	31.7 (5.0)
Stage 2	32.2 (4.67)	28.8 (4.64)	32.0 (5.47)

*FAS* full analysis set, *BMI* body mass index, *MADRS* Montgomery-Asberg Depression Rating Scale, *s.d.* standard deviation

### Efficacy (FAS)

Efficacy analyses are presented in Tables 2–4. Overall, there was no statistically significant difference between either dose of NSI-189 and placebo in terms of reduction of the study primary outcome measure (MADRS) or the HAMD-17. However, the 40 mg dose group demonstrated overall statistically greater reductions in depressive symptoms on two subject-rated scales (SDQ, CPFQ) compared to placebo in the pooled SPCD analyses, and in Stage 2 on the QIDS-SR. The following SDQ items showed statistically significant advantages for NSI-189 40 mg versus placebo (pooled SPCD analyses): low affect (*p* = 0.032), mood responsiveness (*p* = 0.021), being prone to tears (*p* = 0.018), anxiety (*p* = 0.035), ability to make decisions (*p* = 0.047), ability to work (*p* = 0.032), functioning (*p* = 0.002), optimism (*p* = 0.011) and outlook on life (*p* = 0.036). Leaving out the CPFQ items from SDQ did not affect SDQ significance (*p* = 0.040), suggesting non-overlapping effects between depression and cognition symptoms. NSI-189 treatment showed significant improvement in several CogScreen (Table 4), but not Cogstate (Table 5) measures.

**Table 2:** Efficacy analyses (MMRM-FAS): primary outcome measure

	NSI-189 40 mg/day	NSI-189 80 mg/day	Placebo
MADRS (change from baseline)			
Stage 1, mean (s.d.), *n*	−12.6 (11.2), 43	−11.7 (10.8), 43	−10.8 (11.1), 124
REML estimate (95% CI)	−1.8 (−5.6, 1.9)	−1.4 (−5.2, 2.3)	
*p* value, Cohen’s *d*	0.342, −0.17	0.446, −0.14	
Stage 2, mean (s.d.)	−3.7 (7.5), 21	−3.0 (8.1), 22	−2.0 (6.8), 22
REML estimate (95% CI),	−1.8 (−6.4, 2.7)	−1.4 (−5.9, 3.2)	
*p* value, Cohen’s *d*	0.432, −0.25	0.552, −0.19	
Pooled SPCD			
REML estimate (95% CI)	−1.8 (−4.8, 1.1)	−-1.4 (−4.4, 1.5)	
*p* value	0.224	0.344	
MADRS responders			
Stage 1, *N*/*n* (%)	16/43 (37.2)	14/43 (32.6)	41/124 (33.1)
*p* value	0.636	0.962	
Stage 2, *N* (%)	2/21(9.5)	4/22 (18.2)	2/22 (9.1)
*p* value	0.845	0.606	
SPCD *p* value	0.738	0.643	
MADRS remitters			
Stage 1, *N*/*n* (%)	9/43 (20.9)	11/43 (25.6)	23/124 (18.5)
*p* value	0.723	0.331	
Stage 2, *N*/*n* (%)	3/21 (14.3)	4/22 (18.2)	1/22 (4.5)
*p* value	0.145	0.272	
SPCD *p* value	0.134	0.178	

*MMRM-FAS* mixed-effect model repeated measurement-full analysis set, *MADRS* Montgomery-Asberg Depression Rating Scale, *REML estimate* restricted maximal likelihood estimate, *SPCD* sequential-parallel comparison design

**Table 3:** Efficacy analyses (MMRM-FAS): other outcome measures

	NSI-189 40 mg/day	NSI-189 80 mg/day	Placebo
HAMD-17			
Stage 1, mean (s.d.)	−8.5 (7.84)	−8.0 (6.93)	−7.4 (7.26)
REML estimate (95% CI)	−1.0 (−3.5, 1.5)	−0.9 (−3.4, 1.6)	
*p* value, Cohen’s *d*	0.452, = −0.14	0.482, −0.13	
Stage 2, mean (s.d.)	−3.4 (6.10)	−2.9 (5.94)	−0.8 (5.55)
REML estimate (95% CI)	−1.9 (−5.4, 1.6)	−2.2 (−5.6, 1.2)	
*p* value, Cohen’s *d*	0.297, −0.33	0.212, −0.38	
Pooled SPCD			
REML estimate (95% CI)	−1.4 (−3.6, 0.7)	−1.5 (−3.6, 0.6)	
*p* value	0.195	0.152	
SDQ			
Stage 1, mean (s.d.)	−37.3 (33.56)	−31.3 (33.96)	−33.3 (27.63)
REML estimate (95% CI), *p* value	−3.0 (−12.4, 6.4)	−0.7 (−10.0, 8.5)	
*p* value, Cohen’s *d*	0.532, −0.11	0.877, −0.03	
Stage 2, mean (s.d.)	−12.3 (24.73)	1.6 (23.75)	1.1 (19.18)
REML estimate (95% CI), *p* value	−13.4 (−26.3, −0.5*)*	−4.2 (−17.3, 8.8)	
*p* value, Cohen’s *d*	**0.046**, −0.64	0.528, −0.20	
Pooled SPCD			
REML estimate (95% CI), *p* value	−8.2 (−16.2, −0.2)	−2.5 (−10.5, 5.5)	
*p* value	**0.044**	0.543	
CPFQ			
Stage 1, mean (s.d.)	−8.2 (7.61)	−7.0 (7.24)	−5.7 (6.48)
REML estimate (95% CI), *p* value	−1.7 (−3.8, 0.5)	−1.4 (−3.5, 0.7)	
*p* value, Cohen’s *d*	0.133, −0.28	0.191, −0.23	
Stage 2, mean (s.d.)	−3.0 (5.66)	−0.5 (4.49)	−0.8 (4.57)
REML estimate (95% CI), *p* value	−2.1 (−4.9, 0.7)	−0.5 (−3.3, 2.4)	
*p* value, Cohen’s *d*	0.139, −0.47	0.757, −0.10	
Pooled SPCD			
REML estimate (95% CI), *p* value	−1.9 (−3.7, −0.1)	−0.9 (−2.7, 0.8)	
*p* value	**0.035**	0.302	
QIDS-SR (ANCOVA)			
Stage 1, mean (s.d.)	−5.2 (5.50)	−4.7 (5.69)	−5.1 (4.90)
REML estimate (95% CI), *p* value	0.1 (−1.5, 1.8)	0.0 (−1.7, 1.6)	
*p* value, Cohen’s *d*	0.877, 0.03	0.960, −0.01	
Stage 2, mean (s.d.)	−2.4 (3.17)	−1.1 (3.65)	0.2 (4.35)
REML estimate (95% CI), *p* value	−2.5 (−4.8, −0.2)	−1.4 (−3.7, 0.8), 0.210	
*p* value, Cohen’s *d*	**0.040**, −0.68	0.210, −0.39	
Pooled SPCD			
REML estimate (95% CI), *p* value	−1.2 (−2.6, 0.2),	−0.7 (−2.1, 0.6)	
*p* value	0.105	0.293	
CGI-S			
Stage 1			
OLS estimate (95% CI)	−0.07 (−0.47, 0.34)	−0.15 (−0.56, 0.26)	
*p* value, Cohen’s *d*	0.748, −0.05	0.469, −0.07	
Stage 2			
OLS estimate (95% CI)	−0.45 (−0.97, 0.07)	−0.50 (−1.01, 0.02)	
*p* value, Cohen’s *d*	0.092, −0.56	0.059, −0.66	
Pooled SPCD			
OLS estimate (95% CI)	−0.26 (−0.59, 0.07)	−0.32 (−0.65, 0.00)	
*p* value	0.125	0.052	
CGI-I			
Stage 1			
Odds ratio (95% CI)	0.93 (0.50, 1.74)	0.71 (0.38, 1.30)	
*p* value, Cohen’s *d*	0.830, −0.01	0.267, −0.14	
Stage 2			
Odds ratio (95% CI)	0.35 (0.12, 1.07)	0.44 (0.14, 1.36)	
*p* value, Cohen’s *d*	0.065, −0.58	0.153, −0.46	
Pooled SPCD			
Odds ratio (95%CI)	0.57 (0.30, 1.08)	0.55 (0.29, 1.06)	
*p* value	0.086	0.075	

*MMRM-FAS* mixed-effect model repeated measurement-full analysis set, *HAMD17* Hamilton Depression Rating Scale-17 Items, *SDQ* Symptoms of Depression Questionnaire, *CPFQ* Cognitive and Physical Functioning Scale, *QIDS-SR (ANCOVA)* Quick Inventory of Depressive Symptomatology Scale-Self Rated (analysis of covariance), *CGI-S*  clinical global impressions-severity, *CGI-I* clinical global impressions improvement, *SPCD*  = sequential-parallel comparison design;, *REML Eestimate*  = restricted maximal likelihood estimate;, *OLS eEstimate*  = ordinary least- squares estimate;, *95% CI*  = 95% confidence interval;, *s.d.* =  standard deviation

Bold fonts indicate *p* values < 0.05

**Table 4:** CogScreen results

Subtest name	Domain	Measurement (including abbreviation)				
		Accuracy	Speed	Throughput	Process	Feedback
Previous Number	Working memory	PNAACC				PNAItoC, PNAItoI
SAT Arrow Direction	Processing speed	SATADACC	SATADRTC	SATADPUT		
SAT Arrow Color	Executive function	SATACACC	SATACRTC	SATACPUT		
SAT Instruction	Working memory	SATINACC	SATINRTC	SATINPUT		SATIN_ItoI, SATIN_ItoC
						SATINRT_C, SATINRT_I
SAT Discovery	Executive function	SATDIACC	SATDIRTC		SATDIRUL	SATDI_ItoI, SATDI_ItoC
					SATDIFAI	SATDIRT_C, SATDIRRT_I
					SATDIPER	SATDIRTSD_C, SATDIRTSD_I
Symbol Digit Coding	Processing speed	SDCACC	SDCSDRT	SDCCORR		
Symbol Digit Coding Delayed Recall	Delayed memory	SDCDRACC				

	Processing speed				Executive function				Working memory				Delayed memory	
	SATADACC		SATADRTC		SATACACC		SATDIRTSD_I		SATIN_ItoI		SATINRT_I		SDCDRACC	
	40 mg	80 mg	40 mg	80 mg	40 mg	80 mg	40 mg	80 mg	40 mg	80 mg	40 mg	80 mg	40 mg	80 mg
Stage 1														
LS mean^a^ (95% CI)	−0.667 (−2.44, 1.10)	−0.463 (−2.20, 1.27)	0.076 (0.02, 0.13)	0.022 (−0.03, 0.08)	−1.248 (−4.98, 2.49)	0.097 (−3.58, 3.77)	42 (−45, 130)	156 (70, 242)	−0.005 (−0.46, 0.45)	0.129 (−0.32, 0.58)	93 (−255, 443)	314 (−2, 631)	−2.387 (−11.35, 6.58)	−4.345 (−13.20, 4.51)
*p* value	0.458	0.599	**0.009**	0.443	0.511	0.958	0.341	**<0.001**	0.982	0.571	0.594	**0.052**	0.600	0.334
Cohen’s *d*	−0.14	−0.10	0.50	0.14	−0.124	0.010	0.18	0.67	−0.004	0.106	0.16	0.53	−0.099	−0.181
Stage 2														
LS mean^a^ (95% CI)	−2.016 (−6.03, 2.00)	−3.611 (−7.41, 0.19)	0.036 (−0.05, 0.13)	0.023 (−0.06, 0.11)	−5.055 (−10.21, 0.10)	−4.552 (−9.50, 0.40)	45 (−123, 213)	−71 (−235, 91)	0.687 (0.11, 1.26)	0.430 (−0.12, 0.98)	539 (−70, 1148)	384 (−251, 1020)	−24.658 (−39.52, −9.80)	−15.323 (−29.58, −1.06)
*p* value	0.318	0.062	0.418	0.599	0.054	0.071	0.593	0.383	**0.020**	0.125	0.080	0.224	**0.002**	**0.036**
Cohen’s *d*	−0.35	−0.63	0.27	0.17	−0.66	−0.59	0.18	−0.29	0.81	0.51	0.82	0.59	−1.12	−0.69
Combined														
ANCOVA *p*	0.221	**0.051**	**0.034**	0.387	**0.048**	0.150	0.356	0.363	0.064	0.118	0.065	**0.044**	**0.002**	**0.019**

*SATADACC*  SAT-Arrow Direction Accuracy (% accurate response, 100% = perfect), *SATADRTC *  SAT-Arrow Direction Speed (s), *SATACACC*  SAT-Arrow Color Accuracy (% accurate response, 100% = perfect), *SATDIRTSD_I * SAT-Discovery Reaction Time Variability Following Incorrect Response (ms), *SATIN_ItoI* SAT-Instruction Number Incorrect Following Incorrect Response (count), *SATINRT_I* SAT-Instruction Reaction Time Following Incorrect Response (ms), *SDCDRACC* SDC-Delayed Recall Accuracy (% accurate response, 100% = perfect), *LS mean* least-squares mean, *ANCOVA* analysis of covariance

*t Tests with *p* ≤ 0.05

^a^ LS means are from the ANCOVA model for differences between active and placebo groups

Bold font indicates *p* values < 0.05

**Table 5:** Cogstate results

	Test name							
	Detection		Identification		One Card Learning		One Back	
	40 mg	80 mg	40 mg	80 mg	40 mg	80 mg	40 mg	80 mg
Stage 1								
* p* value	0.504	0.237	0.929	0.543	0.870	0.637	0.975	0.767
Cohen’s *d*	0.147	−0.173	−0.060	0.131	−0.127	0.048	−0.042	−0.029
Stage 2								
* p* value	0.499	0.506	0.618	0.627	0.642	0.384	0.172	0.290
Cohen’s *d*	0.112	0.106	0.113	−0.309	−0.145	0.328	0.327	0.239
Combined								
* p* value	≥0.05	≥0.05	≥0.05	≥0.05	≥0.05	≥0.05	≥0.05	≥0.05

### Safety and tolerability (safety dataset)

During Stage 1 there were no discontinuations for NSI-189 40 mg and 80 mg due to intolerance. In contrast, there were seven discontinuations for placebo due to intolerance. The overall discontinuation rate for the first 6 weeks was significantly higher for placebo (*n* = 25, 18.9%) than 40 mg (*n* = 4, 9.1%) or 80 mg (*n* = 1, 2.3%) (*χ*^2^ = 8.749, df = 2; *p* = 0.013). In Stage 2, there was one discontinuation for NSI-189 40 mg, 0 for 80 mg, and 1 for placebo due to intolerance. No subjects randomized to treatment with NSI-189 experienced a serious adverse event during the study. Adverse event rates for those events rated “likely related” or “related” to treatment which occurred with an incidence 2.5% or greater in at least one treatment group are reported in Supplemental Table 2.

## Discussion

The present exploratory study is the second placebo-controlled trial evaluating NSI-189 in patients with MDD. This study was powered to detect a treatment effect size of 0.5 (Cohen’s *d*) or greater for either of the two doses tested (40 mg versus 80 mg) versus placebo, which is somewhat larger than the Cohen’s *d* of 0.31 (CI = 0.27–0.35) which has been reported for antidepressant drugs compared to placebo [42]. In terms of efficacy, neither dose of NSI-189 showed a statistically significant reduction in symptoms during the trial compared to placebo on either of the two traditional clinician-rated depression severity scales: the MADRS and the HAMD-17. However, the 40 mg dose of NSI-189 resulted in significantly greater symptom reduction versus placebo overall on two different subject-rated depression and cognition severity scales (SDQ and CPFQ). In addition significant treatment effects were found for the QIDS-SR for Stage 2, the third self-rated used in the trial. The lack of overall (pooled Stages 1 and 2) significance for the QIDS-SR may be due to the fact that, unlike the SDQ and CPFQ, the QIDS-SR was only administered at baseline and endpoint for each stage (making an MMRM analysis impossible). Based on these findings, the results of the present study are consistent with those reported from the previous MDD trial in which oral NSI-189 was found to be superior to placebo on the SDQ as well as the CPFQ. Consistent with the present study, statistical significance was not achieved on the MADRS in the phase 1b study, though similar effect sizes were reported for these as with the self-report measures of depressive symptoms. From a safety/tolerability standpoint, NSI-189 was relatively well tolerated and no SAEs were reported.

It is worth noting that, in both trials, relatively broad-scope self-report symptom measures of depression and cognition appear to have outperformed traditional clinician-rated measures which sample only a limited subset of possible symptoms MDD patients report in clinical practice, or even when compared to those listed in the DSM-5. Indeed, it has been argued that the MADRS and HAMD-17 were tailored with the particular symptom and side-effect profile of older agents in mind, namely the tricyclic antidepressants. The drawback, however, is that these older scales (developed in the 1950s and 1960s) fail to capture improvement on several key domains including cognition, irritability, reverse neuro-vegetative symptoms, and emotional symptoms specific to atypical depression including mood reactivity and rejection sensitivity. Interestingly enough, statistically significant separation on the SDQ was obtained on symptoms captured by the MADRS/HAMD-17 such as low affect, tearfulness, but also others including anxiety, mood reactivity, ability to make decisions, ability to work, functioning, optimism, and outlook on life. Efficacy, as measured on the CPFQ, reflects improvement in cognition during treatment. If these assumptions are correct, it is clear in our opinion, that relying on new technology to screen for antidepressant drugs in the pre-clinical arena but then testing them with scales developed 50-60 years ago falls short of what is needed to move the field forward. What remains unclear is why the 80 mg daily dose did not demonstrate efficacy on any study measure, save for some measures of objective cognition. Although it is possible that doses higher than 40 mg are not efficacious for the treatment of symptoms of MDD, the previous phase 1b trial did not show evidence for differential efficacy across daily doses of 40, 80, and 120 mg (Figs. 5a, d in the respective publication). Unfortunately, doses lower than 40 mg have not been tested in order to help confirm or refute this hypothesis.

In line with our initial hypothesis as outlined in the introduction of this manuscript, NSI-189 40 mg demonstrated significant changes on multiple domains of cognition measured by CogScreen, including response accuracy on an executive function measure assessing mental flexibility, response speed on a measure of choice reaction time, and accuracy on a measure of delayed recall for symbol digit paired associates. In addition, experimental endpoints evaluating response accuracy and speed following negative feedback (i.e., error signals) also showed significant benefit following NSI-189. Prior research indicates that response to negative feedback may reflect a specific cognitive effect of depression [43]. Delayed memory recall from the CogScreen symbol digit coding test (analogous to the paper digit symbol substitution test) showed particularly high treatment effect size. This test has been shown to discriminate among active pilots apolipoprotein E ε4 carriers from non-carriers younger than age 65 years [44], who are associated with reduced HI engagement during episodic memory tasks as measured by functional magnetic resonance imaging [45]. Given the existing need to develop antidepressants which target cognition and executive function in MDD [18–21], the pro-cognitive effect of NSI-189 independent of MADRS reduction is particularly interesting. However, it should be also noted that because of the overall number of objective tests identified, significant findings must be replicated in future studies.

One limitation of our study is that standard clinical trial inclusion and exclusion criteria were applied and, therefore, it is not possible to extend the study findings to groups of subjects excluded such as those older than age 60 years which, in turn, may be of particular interest due to reductions in HI volume seen with age. Similarly, whether NSI-189 would be effective as an adjunct to standard antidepressants cannot be assessed, since concurrent antidepressants were excluded from the study. Furthermore, as mentioned in the previous paragraph, lower doses than 40 mg were not tested, which would further aid in understanding the dose–response relationship of this compound. Finally, the current study design does not provide information on the longer-term (>12 weeks) safety and tolerability of NSI-189 in MDD. Future studies are needed in order to help answer these important questions, as well as replicate objective cognitive test findings.

In summary, although this SPCD study of NSI-189 failed to show a statistically significant advantage over placebo on the primary outcome measure, all three self-rated measures of depressive and cognitive symptoms showed significant advantages for NSI-189 40 mg daily at some point during the trial. These results replicate those from a previous MDD study. In addition, the 40 mg dose also showed statistical advantages on objective cognitive measures. These results warrant further evaluation of the antidepressant and pro-cognitive effects of this compound.

## Supplementary information

Supplemental Table 1 Study Events

Supplemental Table 2 Adverse Events

Supplemental Figure 1 Consort Diagram
