# Supplementary material for: A phase 2, double-blind, placebo-controlled study of NSI-189 phosphate, a neurogenic compound, among outpatients with major depressive disorder
Source: Mol Psychiatry. 2019 Jan 9;25(7):1569–79. doi: 10.1038/s41380-018-0334-8 (PMC7303010; doi:10.1038/s41380-018-0334-8)
Supplement: Supplementary file 1 — Supplemental Table 1 Study Events [file 41380_2018_334_MOESM1_ESM.docx]

**Supplemental Table 1: Schedule of Activities**

| **Procedure** | Screening Visit  (Day -28 to -14) | Remote CTNI Interview | Visit 1  Baseline  (Day 1) ^2^ | Visit 2  (Day 7) ^2^ | Visit 3  (Day 14) | Visit 4  (Day 21) ^2^  (Phone visit) | Visit 5  (Day 28) ^2^ | Visit 6  (Day 35) ^2^  (Phone visit) | Visit 7  (Day 42) | Visit 8  (Day 49) ^2^ | Visit 9  (Day 56) ^2^ | Visit 10  (Day 63) ^2^  (Phone visit) | Visit 11  (Day 70) ^2^ | Visit 12  (Day 77) ^2^  (Phone visit) | Visit 13  (Day 84) ^2^  or early termination |
| --- | --- | --- | --- | --- | --- | --- | --- | --- | --- | --- | --- | --- | --- | --- | --- |
| Informed Consent | X |  |  |  |  |  |  |  |  |  |  |  |  |  |  |
| Demographics | X |  |  |  |  |  |  |  |  |  |  |  |  |  |  |
| Inclusion/Exclusion Criteria | X |  | X |  |  |  |  |  |  |  |  |  |  |  |  |
| Medical History | X |  |  |  |  |  |  |  |  |  |  |  |  |  |  |
| Psychiatric History | X |  |  |  |  |  |  |  |  |  |  |  |  |  |  |
| Serum Pregnancy Test^1^  Serum Pregnancy Test | X |  |  |  |  |  |  |  |  |  |  |  |  |  |  |
| Concomitant Medications | X |  | X | X | X | X | X | X | X | X | X | X | X | X | X |
| Consumptive Habits |  |  | X |  |  |  |  |  | X |  |  |  |  |  | X |
| **Safety Assessments** | | | | | | | | | | | | | | | |
| Physical Exam | X |  |  |  |  |  |  |  | X |  |  |  |  |  | X |
| Vital Signs | X |  | X | X | X |  | X |  | X | X | X |  | X |  | X |
| Weight | X |  | X |  | X |  | X |  | X |  | X |  | X |  | X |
| Height | X |  |  |  |  |  |  |  |  |  |  |  |  |  |  |
| Urine pregnancy test |  |  | X |  |  |  |  |  | X |  |  |  |  |  | X |
| Urine Screen – Drugs of Abuse | X |  | X |  |  |  |  |  |  |  |  |  |  |  |  |
| Collect Adverse events |  |  | X | X | X | X | X | X | X | X | X | X | X | X | X |
| Safety Labs | X |  |  |  |  |  |  |  | X |  |  |  |  |  | X |
| Electrocardiogram | X |  |  |  |  |  |  |  | X |  |  |  |  |  | X |

| **Procedure** | Screening Visit  (Day -28 to -14) | Remote CTNI Interview | Visit 1  Baseline  (Day 1)^2^ | Visit 2  (Day 7)^2^ | Visit 3  (Day 14)^2^ | Visit 4  (Day 21)^2^  Phone | Visit 5  (Day 28)^2^ | Visit 6  (Day 35)^2^  Phone | Visit 7  (Day 42)^2^ | Visit 8  (Day 49)^2^ | Visit 9  (Day 56)^2^ | Visit 10  (Day 63)^2^  Phone | Visit 11  (Day 70)^2^ | Visit 12  (Day 77)^2^  Phone | Visit 13  (Day 84)^2^  ^or early^ termination |
| --- | --- | --- | --- | --- | --- | --- | --- | --- | --- | --- | --- | --- | --- | --- | --- |
| **Diagnostic and Efficacy Assessments** | | | | | | | | | | | | | | | |
| Cogstate Brief Battery | X |  | X |  |  |  |  |  | X |  |  |  |  |  | X |
| Cogscreen Battery | X |  | X |  |  |  |  |  | X |  |  |  |  |  | X |
| SAFER Interview |  | X |  |  |  |  |  |  |  |  |  |  |  |  |  |
| SCID | X |  |  |  |  |  |  |  |  |  |  |  |  |  |  |
| MADRS | X | X | X |  | X |  | X |  | X |  | X |  | X |  | X |
| HAMD x |  |  | X | X | X |  | X |  | X | X | X |  | X |  | X |
| CGI-S |  |  | X | X | X |  | X |  | X | X | X |  | X |  | X |
| CGI-I |  |  |  | X | X |  | X |  | X | X | X |  | X |  | X |
| ATRQ | X | X |  |  |  |  |  |  |  |  |  |  |  |  |  |
| QIDS-SR |  |  | X |  |  |  |  |  | X |  |  |  |  |  | X |
| SDQ |  |  | X |  | X |  | X |  | X |  | X |  | X |  | X |
| CPFQ |  |  | X |  | X |  | X |  | X |  | X |  | X |  | X |

^1^Required for all women of childbearing potential.

^2^ Post-baseline study visits had a window of +/- 3 days

SCID: structured clinical Interview for the DSM-5; MADRS: Montgomery-Asberg depression rating scale; HAM-D: 17-item Hamilton depression rating scale; CGI-S/I: clinical global impressions - severity and improvement; ATRQ: antidepressant treatment response questionnaire; QIDS-SR: self-rated version of the quick inventory for depressive symptomatology; SDQ: symptoms of depression questionnaire; CPFQ: cognitive and physical functioning questionnaire.
