# Supplementary material for: A phase 2, double-blind, placebo-controlled study of NSI-189 phosphate, a neurogenic compound, among outpatients with major depressive disorder
Source: Mol Psychiatry. 2019 Jan 9;25(7):1569–79. doi: 10.1038/s41380-018-0334-8 (PMC7303010; doi:10.1038/s41380-018-0334-8)
Supplement: Supplementary file 2 — Supplemental Table 2 Adverse Events [file 41380_2018_334_MOESM2_ESM.docx]

**Supplemental Table 2: Adverse Events***

| **STAGE I** |  |  |  |
| --- | --- | --- | --- |
| **ORGAN SYSTEM** Adverse Event Type | Placebo N = 130, N (%) | NSI-189 40 mg N=44, N (%) | NSI-189 80 mg N=44, N (%) |
| **NEUROLOGIC** |  |  |  |
| Headache | 11 (8.5) | 5 (11.4) | 8 (18.2) |
| Somnolence | 4 (3.1) | 4 (9.1) | 1 (2.3) |
| Dizziness | 3 (2.3) | 1 (2.3) | 2 (4.5) |
| **GASTROINTESTINAL** |  |  |  |
| Nausea | 10 (7.7) | 4 (9.1) | 2 (4.5) |
| Diarrhea | 1 (0.8) | 2 (4.5) | 1 (2.3) |
| Dry Mouth | 14 (10.8) | 2 (4.5) | 1 (2.3) |
| Vomiting | 2 (1.5) | 1 (2.3) | 0 |
| Increased appetite | 0 | 2 (4.5) | 2 (1.5) |
| Decreased appetite | 3 (2.3) | 0 | 0 |
| Abdominal Discomfort | 0 | 1 (2.3) | 0 |
| **INFECTIOUS** |  |  |  |
| Nasopharyngitis | 1 (0.8) | 0 | 0 |
| Upper Resp. Infection | 2 (1.5) | 0 | 0 |
| **PSYCHIATRIC** |  |  |  |
| Abnormal Dreams | 4 (3.1) | 1 (2.3) | 3 (6.8) |
| Insomnia | 5 (3.8) | 0 | 1 (2.3) |
| **DERMATOLOGIC** |  |  |  |
| Acne | 0 | 2 (4.5) | 0 |

| **STAGE 2**  Adverse Event Type | **Placebo N= 63, N (%)** | | **NSI-189 40 mg N=61, N (%)** | **NSI-189 80 mg N=64, N (%)** |
| --- | --- | --- | --- | --- |
| Nausea | 2 (3.2) | | 2 (3.3) | 2 (3.1) |
| Headache | 0 | | 0 | 2 (3.1) |
| Weight gain | | 0 | 0 | 2 (3.1) |

*Deemed likely related or related, with an incidence of 2.5% or greater in at least one of the treatment groups in the safety dataset.
