# Supplementary material for: A phase 2, double-blind, placebo-controlled study of NSI-189 phosphate, a neurogenic compound, among outpatients with major depressive disorder
Source: Mol Psychiatry. 2019 Jan 9;25(7):1569–79. doi: 10.1038/s41380-018-0334-8 (PMC7303010; doi:10.1038/s41380-018-0334-8)
Supplement: Supplementary file 3 — Supplemental Figure 1 Consort Diagram [file 41380_2018_334_MOESM3_ESM.pptx]

## Slide 1
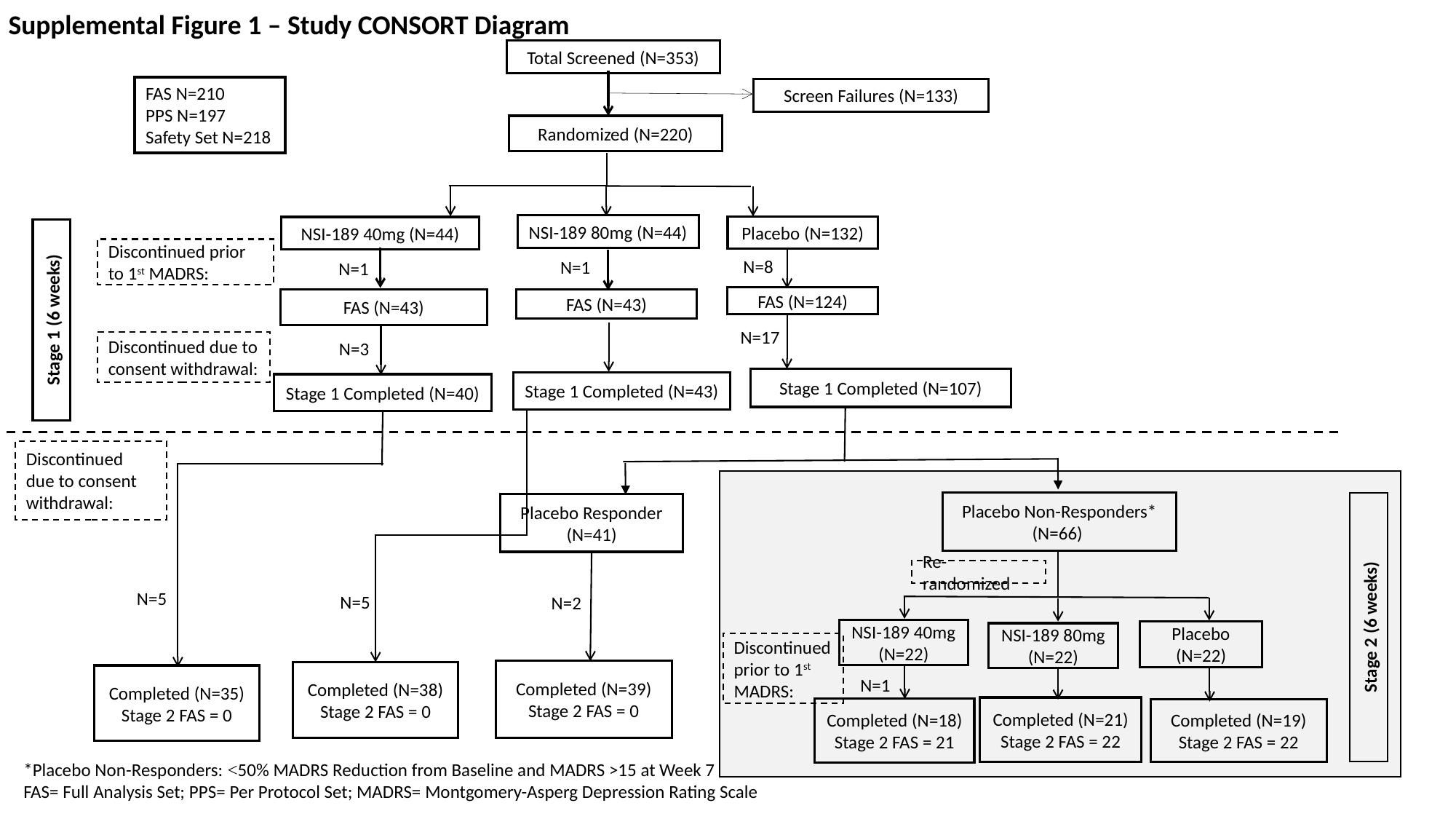

Supplemental Figure 1 – Study CONSORT Diagram
Total Screened (N=353)
FAS N=210
PPS N=197
Safety Set N=218
Screen Failures (N=133)
Randomized (N=220)
NSI-189 80mg (N=44)
Placebo (N=132)
NSI-189 40mg (N=44)
Stage 1 (6 weeks)
Discontinued prior to 1st MADRS:
Stage 1 Completed (N=43)
Stage 1 Completed (N=40)
Placebo Non-Responders* (N=66)
Stage 2 (6 weeks)
Placebo Responder
(N=41)
N=5
NSI-189 40mg
(N=22)
Placebo
(N=22)
NSI-189 80mg
(N=22)
Completed (N=39)
Stage 2 FAS = 0
Completed (N=38)
Stage 2 FAS = 0
Completed (N=35)
Stage 2 FAS = 0
Completed (N=21)
Stage 2 FAS = 22
Completed (N=18)
Stage 2 FAS = 21
Completed (N=19)
Stage 2 FAS = 22
*Placebo Non-Responders: <50% MADRS Reduction from Baseline and MADRS >15 at Week 7
FAS= Full Analysis Set; PPS= Per Protocol Set; MADRS= Montgomery-Asperg Depression Rating Scale
N=8
N=1
N=1
FAS (N=124)
FAS (N=43)
FAS (N=43)
N=17
N=3
Discontinued due to consent withdrawal:
Stage 1 Completed (N=107)
Discontinued due to consent withdrawal:
Re-randomized
N=5
N=2
Discontinued prior to 1st MADRS:
N=1
